# Changes in Diabetes Care and Management Practices during the COVID-19 Pandemic

**DOI:** 10.21203/rs.3.rs-3849240/v1

**Published:** 2024-01-18

**Authors:** Kushagra Vashist, Jennifer K. Frediani, Mary Beth Weber, Mohammed K. Ali, K. M. Venkat Narayan, Shivani A. Patel

**Affiliations:** Emory University; Emory University Nell Hodgson Woodruff School of Nursing; Emory University; Emory University; Emory University; Emory University

**Keywords:** Diabetes management, COVID-19, health services

## Abstract

**Background:**

Evidence suggests diabetes management was negatively impacted early in the pandemic. However, the impact of the pandemic on key healthcare services for diabetes control and diabetes self-management practices is less known. We examined changes in diabetes care and management practices before and during the COVID-19 pandemic.

**Methods:**

Population-based data regarding 4 diabetes-related healthcare engagement and 4 self-management indicators were obtained from adults with diabetes surveyed in 19 US States and Washington DC through the Behavioral Risk Factor Surveillance System. Using logistic regression, we estimated changes in the prevalence of each indicator, overall and by sociodemographic subgroups, before (2019; n = 15,307) and during (2021; n = 13,994) the COVID-19 pandemic.

**Results:**

Between 2019 and 2021, the prevalence of biannual HbA1c tests reduced by 2.6 percentage points (pp, 95% CI :−4.8, −0.4), from 75.4–73.1%, and prevalence of annual eye exams fell by 4.0 pp (−6.2, −2.8), from 72.2–68.7%. The composite indicator of engagement with healthcare for diabetes control fell by 3.5 pp (−5.9, −1.1), from 44.9–41.9%. Reductions in engagement with healthcare were largely seen across sex, age, education, employment status, marital status, insurance status, and urbanicity; and were more pronounced among those aged 18–34 and the uninsured. Reductions in engagement with healthcare were seen in several states, with Delaware and Washington DC reporting the largest decrease. Of self-management behaviors, we only observed change in avoidance of smoking, an increase of 2.0 pp (0.4, 3.6) from 84.7–87.1%.

**Conclusions:**

The pandemic had mixed impacts on diabetes care and self-management. The findings suggest a deterioration of the uptake of evidence-based, preventive health services requiring laboratory services and clinical examination for diabetes control during the pandemic. On the other hand, smoking rates decreased, suggesting potential positive impacts of the pandemic on health behaviors in people with diabetes.

## Background

Diabetes is a chronic condition that requires both continual engagement with healthcare and routine self-management, without which poor glycemic control and diabetes-related complications are more likely^1^. With respect to engagement with healthcare, the American Diabetes Association (ADA) recommends that at minimum diabetes care includes biannual glycated hemoglobin checks from a health professional^2^, annual foot and eye exams^3^. For routine self-management of diabetes, the ADA recommends daily foot checks^3^, regular physical activity,^4^ tobacco avoidance^5^, and participating in educational classes to manage diabetes^6^.

People with diabetes are at particularly high risk for severe outcomes following COVID-19 disease. Simultaneously, diabetes care and self-management in this high-risk group may have been negatively impacted by the pandemic and associated policies to curb viral transmission. Disruptions to health services^7,8^, changes to routine care^8^, and fear of getting severely ill from the virus^9^ may have prevented some individuals living with diabetes from getting the care they need during the COVID-19 pandemic. Lockdowns and social distancing may have impacted diabetes self-management behaviors such as physical activity^10^. Evidence suggests diabetes management was negatively impacted early in the pandemic. For instance, studies have shown a decrease in blood glucose testing^11–13^ and health professional visits for diabetes during the COVID-19 pandemic in the US^11,14^ in select samples. However, the impact of the pandemic on key healthcare services for diabetes control and diabetes self-management practices is less known, especially in population-based studies covering diverse geographies. Furthermore, it is unclear if there was variation in engagement and self-management across different socio-demographic groups, some of whom may have been resilient to the economic impacts of the pandemic.

Identifying the indirect impact of the pandemic on diabetes management and care is critical to understanding the needs and requirements of those living with diabetes as we move into post-public health emergency recovery. Using the Behavioral Risk Factor Surveillance System (BRFSS), we examined changes in four indicators of engagement with healthcare and five indicators of self-management practices for diabetes— before (2019) and during (2021) the COVID-19 pandemic among people living diabetes.

## Materials and Methods

### Data and sample

The BRFSS is a state-based system of health-related telephone surveys that collect data about non-institutionalized US residents’ health-related risk behaviors, chronic health conditions, and use of preventive services. We used publicly available data from the 2019 and 2021 BRFSS adult samples to obtain measures of engagement with healthcare and diabetes self-management and socio-demographic characteristics before and during the pandemic, respectively. The overall response rates for BRFSS 2019 and 2021 were 49.4% and 44.0% respectively. The combined surveys included 112,323 respondents living with diabetes (excluding gestational diabetes) in the District of Columbia (DC) and the 50 US states. The analysis was further restricted to 37,639 individuals with diabetes in states that asked information on diabetes care practices in both 2019 and 2021 samples. Nineteen states—Alaska, Delaware, Illinois, Indiana, Iowa, Kentucky, Maine, Michigan, Minnesota, Montana, New Hampshire, New Mexico, North Carolina, North Dakota, Pennsylvania, Texas, Virginia, Wisconsin, Wyoming, and the District of Columbia (Washington DC), collected data on needed indicators. Among respondents surveyed in states covering the diabetes module, 22% were excluded due to missing data on any outcome and socio-demographic characteristic, resulting in a final unweighted analytic sample size of 29,301 across the two surveys. The distribution of the socio-demographic characteristics in the 1. total BRFSS sample, 2. among all adults with diabetes (excluding gestational diabetes), 3. states that were asked diabetes-specific questions, and 4. our analytic sample is shown in Additional File 1 in the Supplement.

### Variables and description

#### Pandemic status

Data from participants surveyed in BRFSS 2019 were classified as pre-pandemic and data from participants surveyed in BRFSS 2021 were classified as post-pandemic.

#### Outcomes

Diabetes care practices included four indicators of engagement with healthcare and four indicators of self-management of diabetes, all measured as binary variables (yes/no) and derived from the measures proposed by the ADA. Engagement with healthcare included 1. biannual health professional visits for diabetes, 2. biannual HbA1c testing by a health professional, 3. annual foot exams for sores or irritations by a health professional, and 4. having had an eye exam in which pupils were dilated. Self-management of diabetes included 1. daily glucose self-monitoring if on insulin, 2. checking feet for sores or irritations daily, including by family members or friends, 3. participating in any physical activities or exercise in the past 30 days, and 4. avoiding smoking. The BRFSS survey questions for each indicator are shown in Additional File 2 in the Supplement. We further created a composite measure which indicated whether an individual engaged in all applicable practices for engagement with healthcare for diabetes control.

#### Socio-demographic characteristics

Demographic characteristics included sex (male/female), age (divided into five groups; 18–34, 35–44, 45–54, 55–64, and 65 and older), race/ethnicity (as non-Hispanic White, non-Hispanic Black, Hispanic, non-Hispanic Asian, and Other), and marital status (married or living with a partner). Socioeconomic characteristics included educational attainment (classified as high school or less, some college, or 4 or more years of college), urbanicity (metropolitan or nonmetropolitan), employment status (employed, retired, unemployed/not working), and health insurance (yes or no).

#### STATISTICAL ANALYSIS

We first described the distribution of the socio-demographic subgroups by pandemic status. We then described the prevalence of diabetes care practices (both engagement with healthcare and self-management) before and during the pandemic. The change in the prevalence of the outcomes were predicted marginal differences computed between independent samples of US adults before and during the pandemic, defined by survey timepoint, from the logistic regression models^15^. Pre-pandemic served as the reference group. Both unadjusted and adjusted prevalence differences were estimated. Adjusted estimates accounted for age, sex, race, marital status, urbanicity, insurance status, education level, and employment status. Similarly, we examined the prevalence of engagement with healthcare composite before and during the pandemic among each level of the socio-demographic subgroups and by states with data available on diabetes-specific care modules. All data were analyzed using SAS version 9.4 and SUDAAN version 11.0.1, accounting for the complex survey design.

## Results

Socio-demographic characteristics of respondents in states that administered diabetes-care modules before (2019) and during the pandemic (2021) are shown in Table 1. The demographic composition of adults surveyed before the pandemic and during the pandemic was similar. Respondents with self-reported diabetes had a relatively even sex distribution (49.3% female pre-pandemic; 48% female pandemic), a large proportion of those aged 65 years or older (43.0% pre-pandemic; 44.1% pandemic), and a greater share of Non-Hispanic White individuals (67.0% pre-pandemic; 65.4% pandemic).

Table 2 shows the prevalence of diabetes care practices before (2019) and during (2021) the pandemic. Receipt of an annual eye exam dropped from 72.2% (95% Confidence Interval, CI: 70.7, 73.6) before the pandemic to 68.7% (95% CI: 66.9, 70.5) during the pandemic. The adjusted prevalence difference (aPD) between pre- and during pandemic for having an eye exam in the past year was − 4.0 (95% CI: −6.2, −2.8) percentage points (pp) after accounting for differences in socio-demographic characteristics of the two samples. Receiving biannual HbA1c tests reduced from 75.4% (95% CI: 73.9, 76.8) before the pandemic to 73.1% (95% CI: 71.3, 74.7) during the pandemic (aPD= −2.6 pp [95% CI: −4.8, −0.4]). Among self-management practices for diabetes, the prevalence of avoiding smoking increased from 84.7% (95% CI: 83.5, 85.9) before the pandemic to 87.1% (95% CI: 85.9, 88.2) during the pandemic (aPD = 2.0 pp [95% CI: 0.4, 3.6]).

**Figure 1:**
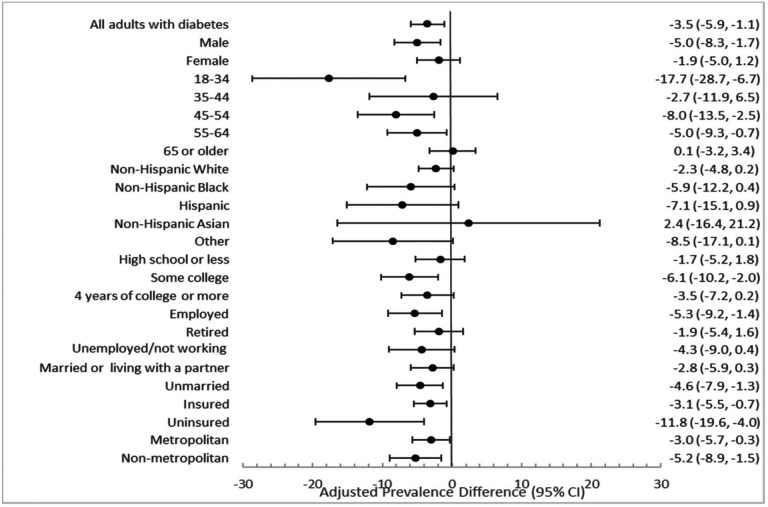
Adjusted prevalence difference (aPD) between 2021 and 2019 for all applicable practices for engagement with healthcare*, among adults with diabetes overall and by socio-demographic subgroups *Consists of all the following healthcare practices- biannual health professional visits for diabetes, biannual HbA1c tests, annual foot, and eye exams ^a^Adjusted for age, sex, race, urbanicity, insurance status, employment status, education level, marital status Error bars represent 95% CI of the adjusted prevalence difference

Between the pre-pandemic to pandemic period, there was a reduction of 3.5 pp (95% CI: −5.9, −1.1) from 44.9% (95% CI: 43.3, 46.5) to 41.9% (95% CI: 40.1, 43.7) in the composite measure of 4 indicators of engagement with healthcare in all people with diabetes (Fig. 1; Additional File 3 in the Supplement). This decrease was consistently observed across 23 of 24 socio-demographic groups, with statistically significant decreases in healthcare engagement from pre-pandemic to during the pandemic in 12 of 24 socio-demographic subgroups. The largest reductions were among those aged 18–34 years (aPD=−17.7 [95% CI: −28.7, −6.7]), from 38.6% (95% CI: 30.6, 47.2) before the pandemic to 23.2% (95% CI: 18.0, 34.1) (Fig. 1; Additional File 3 in the Supplement). Individuals who were uninsured saw the second largest decrease (aPD= −11.8 [95% CI: −19.6, −4.0]), from 23.6% (95% CI: 18.1, 30.2) before the pandemic to 13.0% (95% CI: 8.1, 20.3) during the pandemic.

**Figure 2:**
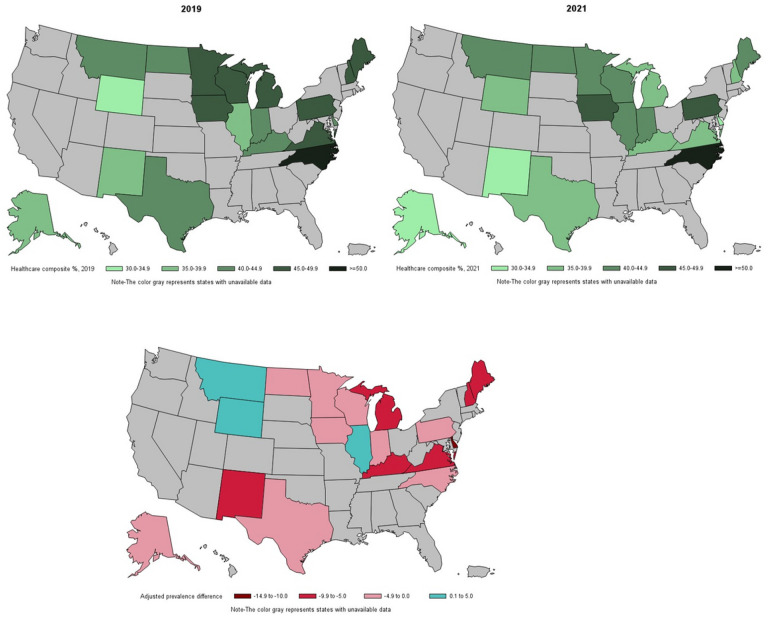
Prevalence of adults with diabetes engaging in all healthcare indicators and adjusted prevalence difference (aPD) between 2021 and 2019 *Adjusted for age, sex, race, urbanicity, insurance status, employment status, education level, marital status

Among 19 US states and Washington DC with data on diabetes-specific care modules, 7 saw a significant decrease in the engagement with healthcare composite measure from pre-pandemic to during the pandemic. These included Delaware, Washington DC, Maine, Michigan, New Hampshire, New Mexico, and Virginia (Fig. 2; Additional File 4 in the Supplement). Delaware and Washington DC saw the largest reduction in the engagement with healthcare (−11.5 [95% CI: −19.7, −3.3] and − 12.8 [95% CI: −24.2, −1.4] respectively). North Carolina reported the highest engagement with health care both pre-pandemic and during pandemic (> 50%). No states experienced a significant increase in engagement with healthcare.

## Discussion

Corresponding to the COVID-19 pandemic, between 2019 and 2021, US adults with diabetes reported reductions in engagement with healthcare, including a 2.6 pp decrease in biannual HbA1c tests and 4 pp decrease annual eye exams. On the other hand, the proportion of people with diabetes who reported not smoking increased while all other self-management practices for diabetes control stayed similar. Reduction in engagement with healthcare was seen across nearly all socio-demographic subgroups and was more pronounced among those aged 18–34 and the uninsured. Similarly, we observed reductions in engagement with healthcare in 18 of 20 states, with Delaware and Washington DC reporting the largest decrease. These findings suggest that the pandemic had a negative impact on uptake of diabetes related health services – including HbA1c testing and eye examination—with little impact on diabetes self-management behaviors at home.

Existing studies lack data on the impact of the COVID-19 pandemic on other specific components of diabetes care, such as annual eye exams; however, this study presents a unique opportunity to understand the ramifications caused by the pandemic on these relatively unexplored components. In our study, we saw a decrease in having an annual eye exam during the pandemic. The limited existing evidence suggests that the pandemic negatively impacted eye exams as part of diabetes care^16^. Diabetic retinopathy screening was also severely impacted by the pandemic, especially in areas of high SARS-CoV-2 transmission^17^. The decline in receiving eye exams is especially concerning due to the importance of regular screening in preventing diabetic retinopathy and severe vision loss^18^.

Unlike annual health exams, there was no change in biannual health professional visits for diabetes. A possible explanation for this is the rise in telehealth during the pandemic, and its potential to replace routine healthcare visits not requiring specialty examinations. Yet, despite no change in routine healthcare visits, there was a reduction in the proportion receiving HbA1c tests. Data from the nationally representative National Health Interview Survey (NHIS) also indicated a decrease in blood glucose testing during the pandemic^13^. In serial cross-sectional NHIS, the prevalence of annual HbA1c checking in US adults with diabetes reduced from 96.8% in 2019 to 94.2% in 2021, suggesting that the drop in glucose checking by health professionals may have extended beyond the first year of the pandemic^12^. The ADA recommends at minimum, biannual HbA1c testing in those with diabetes, and we observed a reduction in biannual HbA1c testing, from 75.9% in 2019 to 74.2% in 2021. Whether receipt of biannual healthcare visits in the absence of HbA1c testing benefits people with diabetes may also be explored in future studies.

Overall healthcare engagement was investigated through a composite of biannual health professional visits for diabetes, biannual HbA1c tests, annual foot, and eye exams. The magnitude of reduction in overall healthcare engagement was largest in younger adults. Those aged 18–34 years reported a 17.7 pp decrease in healthcare engagement, compared with the national average of 3.5 pp. Younger age groups have reported elevated levels of stress and anxiety due to reasons such as poor sleep^19^, and education-related challenges^20^ during the COVID-19 pandemic, which may have contributed to their lack of visitation to a health professional. Additionally, younger people with diabetes have historically more likely been non-adherent to diabetes complication screening guidelines such as retinal screening, and this reluctance to attend screening visits could have been exacerbated during the pandemic^21,22^.

Uninsured adults with diabetes reported an 11.8 pp decrease in engagement with healthcare, the second largest reduction after younger adults. It has previously been shown that uninsured individuals had a lower odds of having a primary care encounter during the pandemic, which may put them at risk for uncontrolled HbA1c levels^23^.

Notably, there were no statistically significant differences in the magnitude of reduction in healthcare engagement by race. Both Non-Hispanic Black and Hispanic race and ethnicity saw a large decrease in engagement with healthcare, although the prevalence between pre and during pandemic was not statistically significant. However, adults classified in the Other race group reported a large statistical decrease, suggesting the pandemic’s negative impact on health services among minority groups that largely comprise this category, including American Indian/Alaskan Native and Multiracial groups.

While the reduction in healthcare engagement was also consistently observed across surveyed states, 7 states reported statistically significant reductions of 5 pp or larger: Delaware, Maine, Michigan, New Hampshire, New Mexico, Virginia, and Washington DC. Further investigation regarding factors contributing to these large declines is warranted.

Our study has several strengths. First, we report key differences in practices for engagement with healthcare that are specific to diabetes, such as receiving biannual HbA1c tests and annual eye exams, pre and during pandemic. Second, our data afforded us the opportunity to examine changes in previously unexplored diabetes self-management behaviors, such as daily foot checks and daily glucose monitoring. Third, we investigated differences in pandemic impacts on diabetes healthcare services by socio-demographic subgroup, addressing major concerns around health equity in the pandemic. Fourth, we reported changes in diabetes healthcare services by US states.

Several limitations of this study should be acknowledged. We had to limit our analysis to the states which had data for the diabetes care and management practices in both 2019 and 2021; of note, is the fact that the study sample did not include states in the Southeastern US, which report the highest prevalence of diabetes^24^. Relatedly, state-level and subgroup analyses among people with diabetes may have been underpowered to detect statistically significant changes. Individuals with the worst controlled diabetes may have died early in the pandemic^25,26^ eliminating them from this analysis. Therefore, our results may have been conservative and underestimate the lack of self-management and engagement with healthcare practices. BRFSS data are self-reported and subject to recall bias. We were not able to distinguish diabetes type.

## Conclusion

Among US adults with diabetes residing 19 US states and Washington DC, we found reductions in having biannual HbA1c tests, annual eye exams, and participation in diabetes self-management education during the COVID-19 pandemic. Reductions were consistent across socio-demographics and across states. The findings suggest a deterioration of the uptake of evidence-based, preventive health services requiring laboratory services and clinical examination for diabetes control during the pandemic. In contrast, most measures of at-home self-management diabetes behaviors remained comparable before and during the pandemic, while smoking rates decreased. Examining the indirect impact of the pandemic on diabetes care is critical in understanding the needs and requirements of those living with diabetes as we move into a post-pandemic environment. If these changes are further sustained, healthcare systems may need to introduce new measures to rehabilitate diabetes care practices in the post-pandemic era.

## Figures and Tables

**Table 1:** Socio-demographic characteristics of adults living with diabetes, pre- and during pandemic

	Pre-pandemic^a^ 2019	Pandemic^b^ 2021
Overall unweighted n	15,307	13,994
Sex		
Female	49.3 (47.7, 50.9)	48.0 (46.2, 49.8)
Age		
18–34	4.2 (3.6, 5.0)	4.3 (3.5, 5.3)
35–44	7.9 (6.8, 9.1)	8.1 (7.0, 9.3)
45–54	17.1 (15.8, 18.4)	17.6 (16.1, 19.1)
55–64	27.8 (26.4, 29.3)	25.9 (24.4, 27.5)
65 and above	43.0 (41.4, 44.6)	44.1 (42.4, 45.9)
Race/ethnicity		
Non-Hispanic White	67.0 (65.2, 68.7)	65.4 (63.5, 67.3)
Non-Hispanic Black	14.2 (13.1, 15.4)	15.7 (14.4, 17.1)
Hispanic	13.9 (12.4, 15.6)	14.6 (12.9, 16.4)
Non-Hispanic Asian	1.8 (1.4, 2.5)	1.7 (1.3, 2.2)
Other	3.1 (2.7, 3.5)	2.6 (2.2, 3.0)
Education		
High school or less	46.5 (44.9, 48.2)	44.5 (42.7, 46.4)
Some college	33.5 (31.9, 35.0)	33.8 (32.1, 35.5)
4 years of college or more	20.0 (19.0, 21.1)	21.6 (20.4, 22.9)
Employment status		
Employed	37.0 (35.4, 38.6)	36.3 (34.5, 38.0)
Retired	37.8 (36.2, 39.3)	39.1 (37.4, 40.8)
Unemployed/not working	25.3 (23.9, 26.7)	24.7 (23.0, 26.4)
Married or living with a partner	57.7 (56.1, 59.3)	59.2 (57.4, 60.9)
Insured	92.7 (91.6, 93.6)	93.8 (92.2, 95.0)
Metropolitan	76.7 (75.6, 77.9)	77.7 (76.3, 79.0)

a BRFSS 2019

b BRFSS 2021

P-value comparing difference in characteristics between adults surveyed pre pandemic and during the pandemic were as follows; sex (p = 0.0916); age (p = 0.4657); race/ethnicity (p = 0.1668); education (p = 0.0527); employment status (p = 0.3561); marital status (p = 0.1373); insurance status (p = 0.1120); urbanicity (p = 0.2894)

**Table 2:** Prevalence of diabetes care and management practices among adults with diabetes, pre- and during pandemic

	Pre pandemic^a^	Pandemic^b^		
Unweighted n	14,972	13,647		
	% (95% CI)	% (95% CI)	PD (95% CI)	aPD^c^ (95% CI)
**Diabetes care practices**				
**Engagement with healthcare**				
Biannual health professional visits for diabetes	75.9 (74.5, 77.3)	74.2 (72.7, 75.7)	−1.7 (−3.7, 0.3)	−1.9 (−3.9, 0.1)
Biannual HbA1c tests	75.4 (73.9, 76.8)	73.1 (71.3, 74.7)	**−2.3 (−4.5, −0.1)**	**−2.6 (−4.8, −0.4)**
Annual foot exam	78.3 (76.9, 79.6)	77.3 (75.7, 78.7)	−1.0 (−3.0, 1.0)	−1.4 (−3.4, 0.6)
Annual eye exam	72.2 (70.7, 73.6)	68.7 (66.9, 70.5)	**−3.5 (−5.9, −1.1)**	**−4.0 (−6.2, −1.8)**
**Self-management**				
Exercise in past 30 days	61.7 (60.1, 63.2)	61.8 (60.0, 63.6)	0.1 (−2.3, 2.5)	−0.4 (−2.8, 2.0)
Not current smoker	84.7 (83.5, 85.9)	87.1 (85.9, 88.2)	2.4 (0.8, 4.0)	2.0 (0.4, 3.6)
Daily foot checks	57.6 (56.0, 59.2)	59.6 (57.8, 61.4)	2.0 (−0.4, 4.4)	2.1 (−0.3, 4.5)
Daily glucose self-monitoring^‡^	87.9 (85.8, 89.7)	89.2 (87.5, 90.7)	1.3 (−1.2, 3.8)	3.3 (−6.1, 12.7)

a BRFSS 2019

b BRFSS 2021

‡ Restricted to adults using insulin (n = 5,159, pre-pandemic; n = 4599, pandemic)

* Consists of all the following healthcare practices- biannual health professional visits for diabetes, biannual HbA1c tests, annual foot, and eye exams

a Adjusted for age, sex, race, urbanicity, insurance status, employment status, education level, marital status

Error bars represent 95% CI of the adjusted prevalence difference

## Data Availability

The datasets generated and/or analyzed during the current study are available on the Centers for Disease Control and Prevention (CDC) website and can be accessed through this link-https://www.cdc.gov/brfss/index.html
